# Drill holes decrease cancellous bone strength: A comparative study of 33 paired osteoporotic human and 9 paired artificial bone samples

**DOI:** 10.1371/journal.pone.0241143

**Published:** 2020-10-29

**Authors:** Marcin Ceynowa, Krzysztof Zerdzicki, Pawel Klosowski, Rafal Pankowski, Marek Roclawski, Tomasz Mazurek

**Affiliations:** 1 Department of Orthopedic Surgery, Medical University of Gdańsk, Gdańsk, Poland; 2 Faculty of Civil and Environmental Engineering, Gdansk University of Technology, Gdańsk, Poland; University Hospital Zurich, SWITZERLAND

## Abstract

This study was designed to compare compressive strength of cancellous bone retrieved from the femoral head in a specimen with and without guide wire hole, with comparison to synthetic bone samples. Femoral heads retrieved from 33 patients who sustained femoral neck fractures and underwent hip arthroplasty were cut into cuboids leaving two matching samples from the same femoral head. Similar samples were prepared from synthetic femurs. One of the matching samples was chosen at random and was drilled with a guide wire for cancellous screws. The uniaxial compression tests of bone blocks were carried out using the Zwick-Roell Z020 strength testing machine. The mean loss of sample cross section area due to drilling was 24%. The force at failure in drilled specimens was significantly smaller by 18% in human (median: 26%) and by 25% in synthetic bone (median 27%). The strength of human specimens was almost 2 times greater, and their stiffness nearly 4 times greater than in synthetic samples. The study shows that the weakening of the bone after drilling is roughly proportional to the loss of sample cross section area. The percentage decrease in strength was similar in human and artificial bone, but human samples were stronger and stiffer. The comparison shows that forces measured in biomechanical studies on artificial bone cannot be directly attributed to humans, but the relative differences in mechanical properties of synthetic samples after some damage may be accurate and resemble that of human bones.

## Introduction

Proximal femoral fractures are one of the most common fractures treated surgically. The common denominator in surgical technique of fixation of these fractures, both intracapsular and extracapsular, is the placement of a large diameter cannulated screw or blade in the cancellous bone of the femoral head. Their correct placement is important as misplaced implants tend to destabilize, especially in intertrochanteric fractures [1, 2], less so the in treatment of femoral neck fractures with cannulated screws [3].

The placement of the implant into the femoral head is ensured by the insertion of a guide wire under fluoroscopic guidance. Misplaced wires are corrected until satisfactory position is achieved, and the final implant is inserted [4]. Every insertion of the guide wire creates a canal in the bone, often in the direct proximity to the final screw, what raises concerns about weakening of the bone [5].

It has been well recognized that screw holes in cortical bone are sites of increased fracture incidence [6], what has been proved biomechanically for predominantly cortical bone [7, 8], but not for cancellous bone. This study was designed to compare the relative compressive strength of paired cancellous bone specimens retrieved from the femoral head with and without guide wire hole. A comparison of purely cancellous bone specimens has not been investigated before in similar intact and drilled samples. The aim was to recreate the compression of the cancellous bone that occurs in the femoral head between the lag screw and the dense subchondral bone. The holes simulate additional drill holes performed during guide wire positioning or small bone cysts around the lag screw.

Moreover, the comparison of compressive strength was made between human and artificial cancellous bone, as synthetic bone models are commonly used to evaluate implant stability [9–11]. Our hypothesis was that guide wire drill holes decrease the strength of cancellous bone samples. Moreover, the artificial bone samples should have similar force at failure, stiffness as well as the decrease of those parameters after drilling as the human samples.

## Materials and methods

### Materials

Femoral heads were retrieved from 38 consecutive patients (30 female, 8 male) who sustained femoral neck fractures and underwent a standard bipolar hip arthroplasty in the course of their treatment. They were operated on within 72 hours from injury. Mean age was 80 (62–96) years old (SD = 10.4). Femoral neck fractures sustained in males above 65 years of age and in females above 50 years of age meet the diagnostic criteria of osteoporosis, according to national guidelines [12]. The femoral head retrieval did not affect decision making in their treatment and did not change the course of the surgical procedure. All patients gave their consent to participate in the study. Femoral heads were fresh frozen right after surgery. Patients with suspected metastatic neoplasms, arthrosis, rheumatoid arthritis or any other condition that could affect bone quality were excluded from the study. This study was approved by the Independent Bioethics Committee at the Medical University of Gdansk, Poland (issued 21.05.2018, NKBBN/228/2018).

For synthetic cancellous bone samples, Synthetic osteoporotic left femora (LD2350.01, cortical low density/soft cancellous bone, Synbone AG, Neugutstrasse 4, 7208 Malans, Switzerland) were used.

### Sample preparation

**Fig 1:**
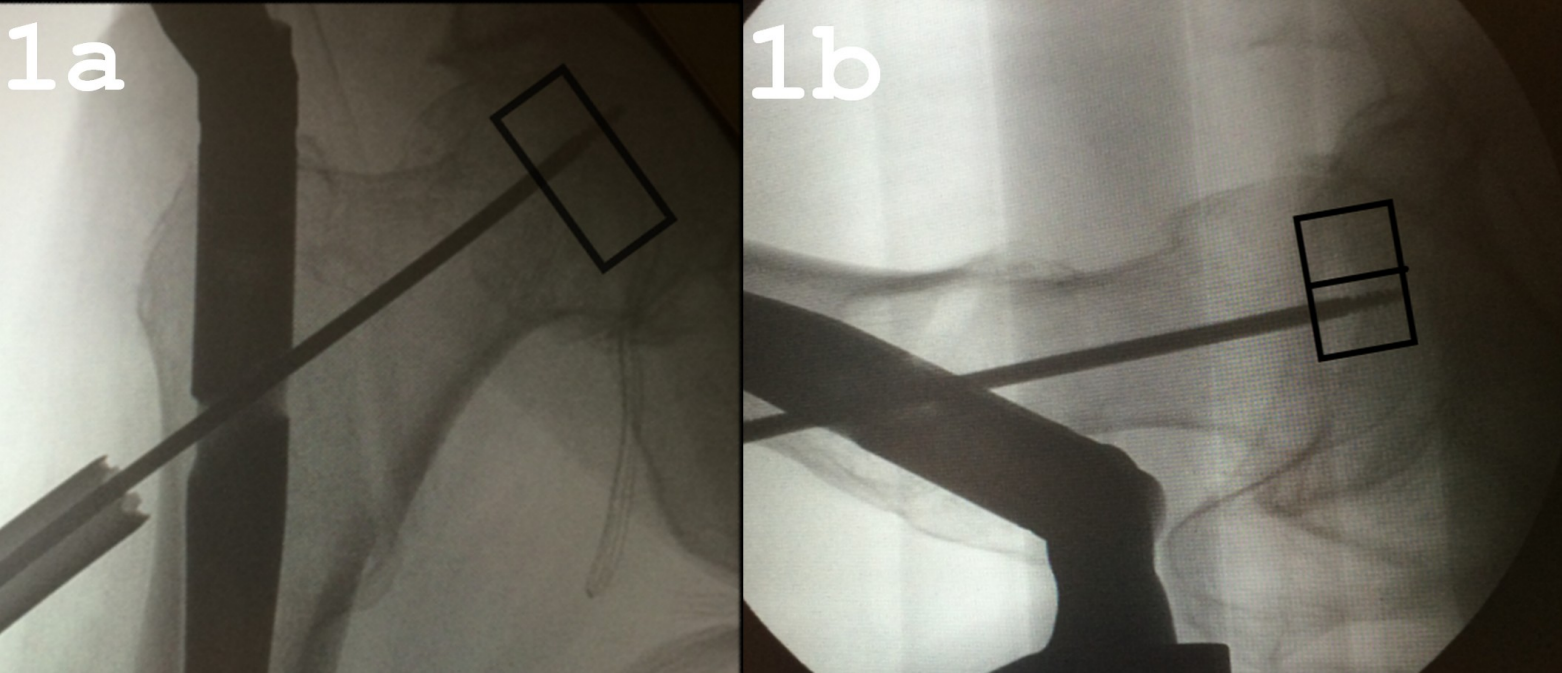
The intraoperative radiographs of intertrochanteric fracture fixation. The shapes drawn in the radiographs show the relative size, shape and direction of the cut bone samples as well as the direction of the hole drilled with the guide wire. (a) anteroposterior view. (b) axial view.

The femoral heads were cut into cuboids using custom made templates with a surgical oscillating saw. The cuboid was then cut in half along the sagittal plane using templates, leaving two matching samples, an anterior and a posterior, from the same femoral head. The size of the cuboid was described as follows: size 1 is the size of the base of the cuboid resulting from the cut in the sagittal plane, size 2 is the length of the base in the coronal plane, and size 2 is the longest side of the cuboid that was directed supero-inferiorly with regard to the long axis of the patients’ body (the sample height) to ensure that the force applied to the sample would have the same vector as hip contact forces for a standing patient. One of the matching samples was chosen at random from each pair. It was drilled with an original 3.2 mm partially threaded guide wire for cancellous screws used in femoral neck osteosynthesis (Ansis III Threaded Guide Wire, Stryker GmbH). Fig 1 show the relative size, shape and direction of the cut bone samples and the direction of the drill hole was directed as it would have been during actual surgery (Fig 1A and 1B).

The synthetic bone samples were prepared in a similar manner from the foam that forms the cancellous bone in femoral head.

After sample preparation a set of 33 pairs of samples of human and 9 pairs of synthetic bone were available for further investigation in this study. The size of the samples as well as their weight were then recorded and the apparent density was calculated from the volume and weight of the sample (S1 File).

The mean percentage loss of cross-sectional area due to drilling was estimated as follows:

Mean loss = (size 1 x 3.2 mm)/(size 2 x size 3) x 100%

The samples were compared statistically (Wilcoxon signed-rank test for comparison of the samples) to ensure that the evaluated physical parameters were similar in both sample groups.

Before testing, human bones were bathed in 37°C saline for 10 minutes after cutting to achieve body temperature. The samples were not cleared of the bone marrow to make sure that the bone resembles as closely as possible its intracorporeal state.

### Mechanical testing

**Fig 2:**
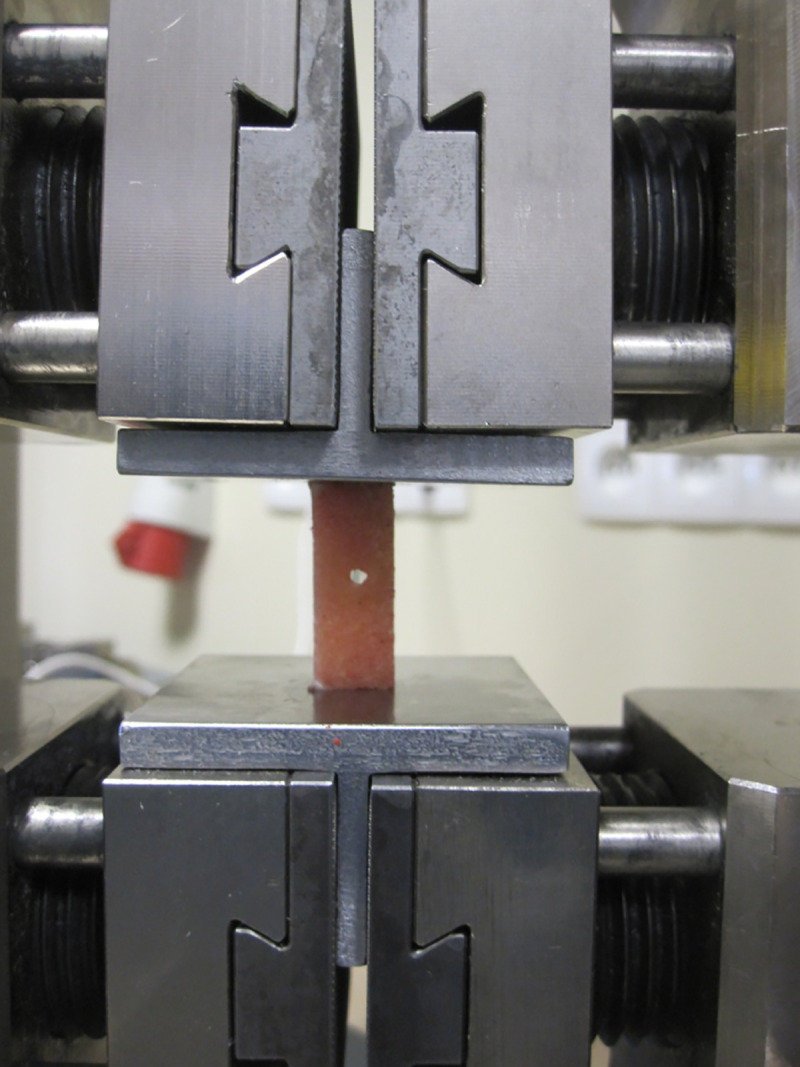
Bone sample fixed to custom-made inserts at Zwick-Roell Z020 testing machine.

The uniaxial compression tests of bone blocks were carried out at room temperature using the Zwick-Roell Z020 testing machine (Fig 2).

**Fig 3:**
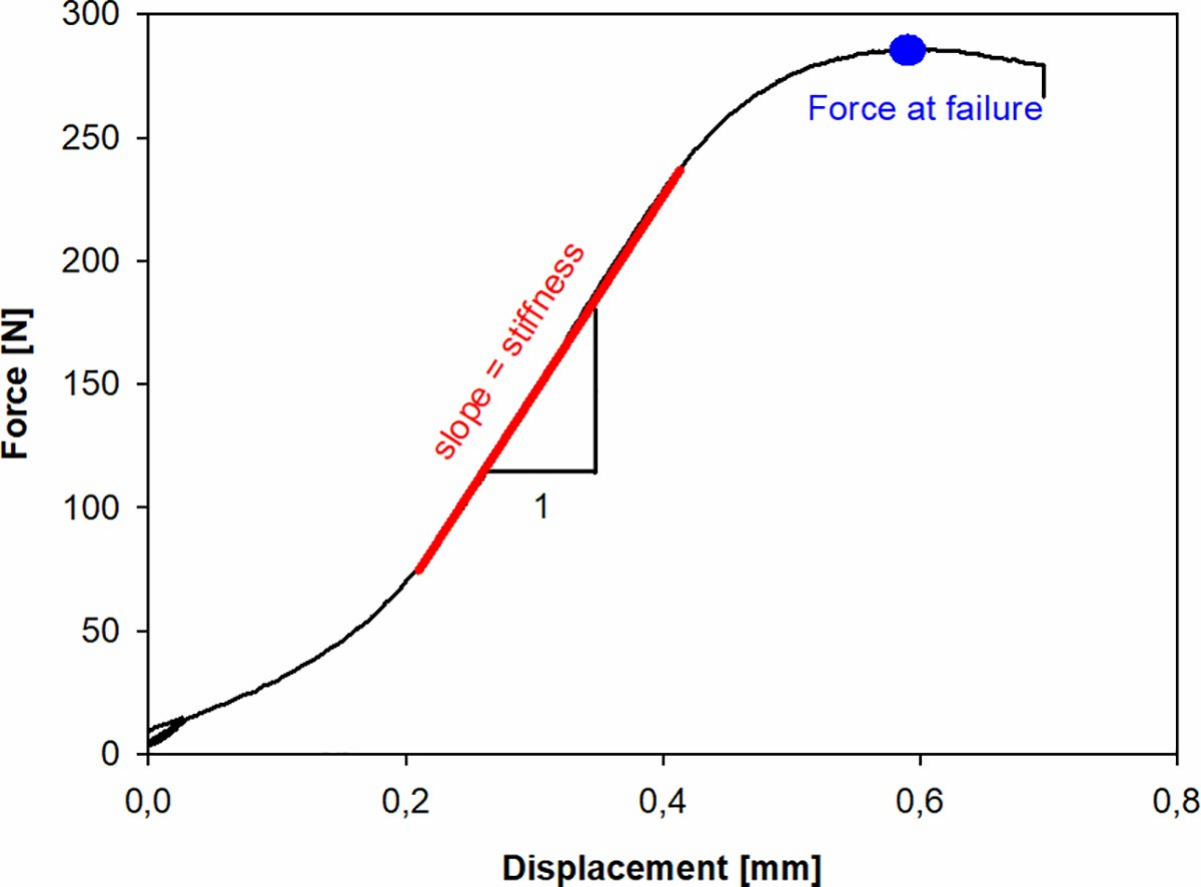
Typical result of the compression test for undrilled sample. The force at failure and stiffness parameters are shown.

The grips separation and actual force were recorded during the test. In the preloading protocol, 3 loading-unloading cycles of the strain range 0–0.1% were applied. The compression loading with the strain rate of 0.5 1/s was continued until the failure of the specimen (Fig 3). A small toe effect being a consequence of adapting of the cuboid samples between testing machine grips and adaptation of the testing machine at the beginning of the experiment, was ignored in further analysis [13].

The results were not recalculated to get their representation in terms of stress and strain relations, as the half of samples were drilled and therefore area of their cross section was not constant along the whole specimen length. The whole bone blocks of exactly same sizes in paired samples, and of slightly differing sizes between pairs, were compared. Consequently, the only reliable measures taken for further analysis were the force at failure and stiffness of the sample.

The highest load level obtained before fracture of the specimen was defined as the force at failure. Stiffness was calculated as the slope of the linear part of the force-displacement curve with skipping the very initial part of the curve, where the toe effect occurred (S1 File).

The percentage difference between the mean values *X* of the obtained characteristics for the drilled and not-drilled samples was calculated as follows: *Difference = (X*_*NOT DRILLED*_*−X*_*DRILLED*_*) / X*_*NOT DRILLED*_ × 100%

Statistical analysis was performed using Statistica PL v. 13.3 software. The Wilcoxon Signed-Rank Test was used for statistical comparison. Results were considered statistically significant with *p* < 0.05.

## Results

The comparison of physical parameters of bone samples are shown in Tables 1 and 2. There were no significant differences regarding size of the sample, weight, volume and density.

**Table 1:** Physical parameters of the human bone samples (n = 33 pairs).

	Not drilled sample (n = 33)	Drilled sample (measurement before drilling) (n = 33)	Statistical comparison (Wilcoxon signed-rank test)
Size 1 (mm) ^a^	Mean: 14.1 (SD = 1.7)	Mean: 14.2 (SD = 1.6)	p = 0.7
	Median: 13.6	Median: 13.9	
	Range: 11.3–17.6	Range: 10.8–17.5	
Size 2 (mm) ^a^	Mean: 12.1 (SD = 1.8)	Mean: 12.1 (SD = 1.8)	p = 0.2
	Median: 12.6	Median: 12.4	
	Range: 7.3–15.1	Range: 7.8–14.5	
Size 3 (mm) ^a^	Mean: 27.1 (SD = 4)	Mean: 26.9 (SD = 3.8)	p = 0.4
	Median: 27.4	Median: 27.6	
	Range: 19–32.8	Range: 18.3–32.7	
Weight (g)	Mean: 4.8 (SD = 1.3)	Mean: 4.8 (SD = 1.5)	p = 0.1
	Median: 4.7	Median: 4.7	
	Range: 3.1–8	Range: 2.8–8.5	
Volume (cm^3^)	Mean: 4.7 (SD = 1.2)	Mean: 4.6 (SD = 1.3)	p = 0.2
	Median: 4.8	Median: 4.6	
	Range: 2.9–7.7	Range: 2.5–7.6	
Density (g/cm^3^)	Mean: 1 (SD = 0.06)	Mean: 1 (SD = 0.08)	p = 0.2
	Median: 1	Median: 1.1	
	Range: 0.9–1.1	Range: 0.9–1.2	

^a^ Size 1 and 2 are the lengths of the base of the sample, and size 3 is the height of the sample.

**Table 2:** Physical parameters of the synthetic bone samples (n = 9 pairs).

	Not drilled sample (n = 9)	Drilled sample (measurement before drilling) (n = 9)	Statistical comparison (Wilcoxon signed-rank test)
Size 1 (mm) ^a^	Mean: 13.3 (SD = 0.5)	Mean: 13.4 (SD = 0.4)	p = 0.5
	Median: 13.4	Median: 13.3	
	Range: 12.5–14.1	Range: 12.9–14.2	
Size 2 (mm) ^a^	Mean: 13 (SD = 0.4)	Mean: 13.1 (SD = 0.6)	p = 0.5
	Median: 13.4	Median: 12.9	
	Range: 12.53–13.7	Range: 12.5–14.5	
Size 3 (mm) ^a^	Mean: 24.7 (SD = 1.2)	Mean: 25.1 (SD = 0.4)	p = 0.8
	Median: 24.7	Median: 25.2	
	Range: 22.8–27.1	Range: 24.4–25.5	
Weight (g)	Mean: 0.62 (SD = 0.06)	Mean: 0.62 (SD = 0.08)	p = 0.4
	Median: 0.6	Median: 0.62	
	Range: 0.5–0.7	Range: 0.5–0.8	
Volume (cm^3^)	Mean: 4.26 (SD = 0.3)	Mean: 4.4 (SD = 0.2)	p = 0.8
	Median: 4.31	Median: 4.4	
	Range: 3.7–4.6	Range: 4.1–4.7	
Density (g/cm^3^)	Mean: 2.67 (SD = 0.4)	Mean: 2.7 (SD = 0.4)	p = 0.6
	Median: 2.63	Median: 2.7	
	Range: 2.1–3.2	Range: 2.1–3.6	

^a^ Size 1 and 2 are the lengths of the base of the sample, and size 3 is the height of the sample.

The estimated mean loss of sample cross section area due to drilling was 25% (median 24%, SD = 5.3) for human bone and 24% (median 24%, SD = 0.73) for synthetic bone.

The strength characteristics of the drilled and not drilled human bone samples are presented in Table 3. The results for synthetic bone are presented in Table 4. The differences between the force at failure were statistically significant, but the comparison of sample stiffness were not.

**Table 3:** Compression test results of human bone samples with definition of mechanical characteristics analyzed in the study (n = 33).

Sample	Force at failure (N)	Stiffnes (N/mm2)
Not drilled	Mean 512.5	Mean 863.2
	Median 446	Median 805.8
	Range 190–1420	Range 107.5–2141.6
	SD 263.24	SD 509.56
Drilled	Mean 422.4	Mean 779.9
	Median 330	Median 698.7
	Range 81–1092	Range 124.9–2626.1
	SD 265.4	SD 567.17
Difference (%)	Mean 18%	Mean 9.8%
	Median 26%	Median 13.3%
Wilcoxon signed rank test	p = 0.04	p = 0.21

**Table 4:** Compression test results of artificial bone samples with definition of mechanical characteristics analyzed in the study (n = 9).

Sample	Force at failure (N)	Stiffnes (N/mm2)
Not drilled	Mean 252.6	Mean 215.9
	Median 253.2	Median 225.1
	Range 187–338	Range 127–306
	SD 51.8	SD 57.8
Drilled	Mean 182.9	Mean 173.3
	Median 182.6	Median 179.6
	Range 146–256	Range 121–243
	SD 33.6	SD 32.9
Difference (%)	Mean 25%	Mean 19.5%
	Median 27%	Median 20%
Wilcoxon signed rank test	p = 0.04	p = 0.21

## Discussion

The successful treatment of proximal femoral fractures depends, among other things, on correct positioning of the implant. In proximal femoral fractures, optimal placement of the guide wire for the lag screw is mandatory to achieve good results [1, 2]. Often, multiple trials are needed to introduce it correctly [4]. It seems safe to assume that multiple drill holes around the final screw or blade of an implant may weaken the bone and lead to the more common screw cutout than when the bone around the implant is intact.

This study has shown human cancellous bone weakening after drilling of a single hole in a bone specimen. Drilling eliminates a portion of the cancellous bone from the transmission of the force applied to the bone and therefore weakens it. The mean and median force at failure are similar to the loss of cross section area of the sample due to drilling. This suggests that the loss of strength of cancellous bone due to drilling may be roughly proportional to the loss of cross section area. The major drawback of this study however is that the effect of the drill hole would be dependent on the size of the bone specimen, since the diameter of the guide wire is constant. It is safe to presume that the smaller the specimen, the greater the impact of the 3.2 mm hole in the bone [14].

In literature, the effect of weakening of the cancellous bone was not studied before in the past the way it is done in this study. In studies of whole bone specimens (cortico-cancellous bone) the decrease in the force to failure in drilled specimens is between approximately 20% to 40%. The fractures occur through the drill holes [7, 8, 15, 16], but the tests were performed as a three-point bending test or a torsional strength test, unlike the current study. Comparisons between cortical and cancellous bone fracture models after drilling should be cautious. Cortical bone is on average 20–30% stronger than cancellous bone, as well as there are differences in elastic modulus and yield strain between those two types of bone [17]. Moreover, there are obvious anatomical differences between whole bone models and a pure cancellous bone sample.

The effect of weakening of the cancellous bone in previous studies is not as obvious as shown for cortical bone. The surprisingly high “safe” number of drill holes (between 14 and 40) found in a recent final element analysis study suggests that a femoral head cancellous bone can withstand multiple drilling attempts without compromising its strength [18]. After anterior cruciate ligament reconstruction acute fractures usually, but not always occur through the drilled canals [19, 20]. In an investigation of composite femur bones, all specimens failed through the femoral canals of the double-bundle ACL reconstruction technique, but in the single bundle technique the fracture patterns were similar to the intact groups and only half occurred through the drill canal [21]. In a similar study of tibial fractures, it was found that load to failure was similar in all groups (intact, single bundle and double bundle reconstructions) [22]. In a biomechanical study of the core decompression of an equine navicular bone, which is predominantely cancellous, it was found that the presence of unicortical bone canals significantly decrease the strength of bone by about 20% [23], what is similar to the current study. However, the size and number of drill holes did not affect the strength of the drilled specimens.

The studies mentioned above suggest that the weakening of the bone due to drilling may not be directly proportional to the size and number of drill holes, and therefore to the volume of the bone stock lost because of drilling. The fact that the loss of strength of cancellous bone that is roughly proportional to the loss of cross-section area of the sample is a rather unexpected finding in this study.

In the current study, no fracture occurred through the drill hole as was expected considering other biomechanical studies [7, 8, 15, 16]. This indicates that failure occurred rather by microfractures of the bone trabeculae of the specimens. It must be noted, however, that in the current study a purely compressive force was applied, and not a torsional or bending force as in others. A compressive force would rather crush the specimen, while a torsional or bending force would fracture the specimen through the point of least resistance, that is, through the hole in the bone.

There are no biomechanical studies that evaluate the human cancellous bone strength after drilling without any cortical component. The implants used in the proximal femoral fractures are fixed in a cancellous bone, and the screw migration occurs within the femoral head, without a typical fracture of the femoral head [1]. Therefore for the purpose of this study, there was a need to examine the cancellous bone without the surrounding cortex.

This study attempts to resemble true in vivo conditions as closely as possible, but generally the tests used to determine the effect of a hole in a bone use a simplified protocol. In true clinical setting, the femoral head is compressed cyclically in changing force vectors because of femoral head rotation in the acetabulum [24]. The site of compression in a fixed intertrochanteric fracture is between the articular surface and the femoral head implant, which is commonly round and threaded, or has several blades. This simplification is another major limitation of the study. In our study, we used a compression test, as was used in testing femoral head cancellous bone samples [9, 17, 25], as the best possible approximation of the forces acting on the cancellous bone in this region. Care was taken to use paired specimens of the same shape, size and origin, and the drilling was assigned to one of the paired specimens randomly [7, 15]. The bone in the anterior and posterior part of the femoral head have the same mechanical properties [26]. The number of paired specimens is similar to other studies [8, 15, 25]. The direction of force in this study is different than the principal compressive region or the main anatomical trabecular direction to ensure similar force direction as occurs in vivo between the hip contact forces of a standing patient and the implant screw.

This study has shown statistically significant cancellous bone weakening after drilling of a single hole in a cancellous bone specimen. The effect of weakening cancellous bone by a guide wire hole cannot be considered yet as consistent as the effect of screw holes in the cortical bone, but should be taken into account in proximal femoral fracture surgery. Further studies, however, are needed to weigh the value of ideally placed implant against weakening of the bone with guide wire holes created by multiple attempts to insert it perfectly. This study suggests that drill holes in the femoral head weaken the bone stock, what may compromise the stability of the fixation. Moreover, since it has been found in other studies that the optimal position of the screws in intracapsular femoral neck fractures has no significant influence on the outcome [3], the authors of the current study no longer attempt multiple times to place the screws in a perfect configuration fearing that multiple drill holes around final implants may weaken the fixation strength.

The synthetic bone model, that was similar in size and shape, shows comparable similarities between loss of force at failure and loss of cross section area as human bone. However, force at failure of human bone is almost twice that of the synthetic bone, and stiffness is approximately 4 times greater in human bone. This shows that biomechanical experiments performed on a synthetic bone cannot be directly translated to human bone, when it comes to absolute measures of the force to failure or stiffness, but relative loss of strength in both types of bones that sustained some kind of damage (fracture, cyst, or implant placement) may be comparable between human and synthetic bone. The experiments performed on synthetic bone probably give an accurate view of the mechanical properties of the fractures or implants compared in biomechanical studies, that is an implant that is inferior in synthetic bone studies most likely will also be inferior in the human bone. However, the magnitudes of forces recorded in synthetic bones may not be accurate in human bones or in patients.

Other studies show some conflicting results regarding mechanical parameters of human v/s synthetic cancellous bone. The screw pullout force, which is probably the only mechanical parameter that can be attributed solely to cancellous bone, was found to be similar to natural bone in one study [27], nearly 4 times greater with standard synthetic bone [28], or exhibited only 40% of pullout strength of natural bone when an osteoporotic type of artificial bone was used. A study that compared stability of identical fracture fixations between human and standard synthetic bone showed that the artificial samples were significantly more stable [10]. Those differences between types of bones and studies can be attributed to the type of material used for comparison. The human bones may have a different degree of osteoporosis and also different types of artificial bones are used (standard v/s osteoporotic type, as well as different generations from different manufacturers). In this study, we used a new type of osteoporotic bone, and compared it to femoral heads from elderly patients who sustained femoral neck fracture, what meets the criteria for osteoporosis [12]. This study, as well as previous studies suggest that a perfect replacement for human bone in mechanical studies has not been found, but the relative differences found in those studies between samples are most likely accurate.

In scientific research, this study supports a good scientific practice to prepare a control group sample (for example, a perfectly reduced fracture fixed with a standard implant, comparable with other studies), and compare differences in force parameters of the other tested groups relative to the standard control group. The absolute values of force to fracture (in N) should be considered with great caution when comparing to other studies, because mechanical properties may vary between artificial bones of different types or between bones from different manufacturers.

The findings of this study support the hypothesis that drill holes decrease the strength of cancellous bone, moreover, the decrease is roughly proportional to the loss of cross section area of the sample caused by the drill hole. The comparison between human and artificial bone shows that forces measured in biomechanical studies on artificial bone are different than in human samples and cannot be directly attributed to humans, but the relative differences in mechanical properties of fractured and fixed synthetic samples may be accurate and resemble that of human bones.

## Supporting information

S1 File Data file. (XLSX)
